# Adaptation and changing phenotypes through transgenerational epigenetics

**DOI:** 10.1080/15592294.2025.2460246

**Published:** 2025-08-13

**Authors:** Lon J. Van Winkle, Rebecca J. Ryznar, Philip M. Iannaccone

**Affiliations:** a Department of Biochemistry, Midwestern University, Downers Grove, IL, USA; b Department of Medical Humanities, Rocky Vista University, Parker, CO, USA; c Molecular Biology, Department of Biomedical Sciences, Rocky Vista University, Parker, CO, USA; d Departments of Pediatrics and Pathology, Northwestern University’s Feinberg School of Medicine, Chicago, IL, USA

**Keywords:** Epigenetics, transgenerational, adaptation, phenotype, evolution, environment

## Abstract

In this article collection, we describe how noncoding epigenetic changes in DNA are transmitted across multiple generations in eukaryotic organisms including plants and animals. And such environmentally induced biochemical alterations of DNA and histones result in profound changes in gene expression. In plants and invertebrate animals, transgenerational epigenetic inheritance has been well documented, and it continues to be substantiated in humans and other vertebrates. These exciting new discoveries have profound consequences for changing as well as maintaining phenotypes expressed by various life forms and, thus, the changes likely contribute to evolution. And in a more practical way, such studies are very important because of the likely transgenerational inheritance of diseases and disorders, such as type 2 diabetes mellitus and obesity.

Epigenetic studies provide insight into the mechanism by which exposure to toxins and other environmental experiences produce undesirable as well as desirable phenotypes in plants, animals, and other eukaryotic organisms. Furthermore, these exciting new discoveries have profound consequences for changing as well as maintaining phenotypes expressed by these life forms. Especially in the field of transgenerational epigenetics, where non-DNA sequence-based alterations are transmitted across generations, such modified phenotypes may be subject to environmental selection. But transgenerational epigenetic inheritance is unlike parental or inter-generational effects where, for example, exposure of mammalian embryos to toxic chemicals in utero alters their sperm or egg cells. Rather, transgenerational epigenetic inheritance occurs when biochemical changes in DNA associated histone proteins or DNA CpG dinucleotide methylations persist without the environmental exposures that led to the epigenetic changes in the first place. Many of these phenomena may allow living organisms not only to adapt to environmental conditions but also to transmit information to future generations that may be beneficial to their survival in challenging environments.

Initial studies of the role of DNA methylation in early embryonic development focused on mechanisms of imprinting, which by selective gene silencing marks the paternal origin of alleles and establishes correct gene dosage for maternal genes. The link between methylation state and imprinting motivated experiments in transgenic mice to establish mechanisms leading to the discovery of irreversible methylation in some of the genes involved [1,2]. This was a surprising contrast to other mammalian DNA methylation events that are reversible and suggested the possible permanence of gene expression status, hence heritability in those genes that did not demethylate. The genome is demethylated in primordial germ cells and then remethylated at the time of embryo implantation [3]. This process is thought to be required to reset the imprints and reactivate genes required for correct development. However, as the transgenic and other experiments show, it is not a complete process and specific genes have been shown to not reset. These experiments demonstrated transgenerational stability of methylation patterns, at least of certain genes in transgenic mice. In the decades since, there has been intensive interest in the stability of epigenetic marks, their role in inheritance, how they can be induced or changed, and the health effects of these modifications.

Nevertheless, in this Article Collection, Khatib and associates [4] challenge the notion that transgenerational epigenetic inheritance has been shown to occur in mammals. They reviewed 80 articles in the literature that claimed to demonstrate transgenerational epigenetic inheritance in mammals. However, most of the articles did not report evidence needed to demonstrate, unequivocally, that such inheritance has occurred. The pertinent criteria for such evidence included inheritance of the same epimutations across generations, gene expression changes in subsequent generations, and testing of germ cells for the epimutations in each generation [4]. In contrast, Fitz-James and Cavalli [5] reviewed several papers not included in the 80 articles reviewed, but which do provide considerable evidence for transgenerational epigenetic inheritance in mammals. For example, Thorson et al. [6] found that exposure of gestating rats to plastic-derived compounds led to sets of DNA methylation biomarkers for specific transgenerational diseases in sperm of affected members of the F3 generation. These diseases included testis, kidney, and multiple (two or more) diseases. Moreover, the epigenetically modified genes identified in this study had been shown in prior studies to be linked to each specific disease.

Similarly, in this article collection, Nicholls et al. reviewed very interesting possible protective transgenerational epigenetic changes associated with diet [7]. In the process, they cited a paper – also not included among the 80 articles reviewed in reference [4] – showing epigenetic changes in neural stem and progenitor cells of descendants of F0 female mice fed a high fat diet [8]. Importantly, these epigenetic changes persisted even in the F3 generation of mice despite the consumption of a standard diet by generations of mice that followed F0 [8].

In another review article in this collection [9], an even more complex possible mechanism of transgenerational epigenetic inheritance in mammals emerges from the papers cited. For example, Nilsson and associates (2023) report that – after F0-F3 sequential exposures to various toxicants – F0-F3 sperm DNA methylation regions showed little overlap [10]. This result indicated to the authors that the pertinent epigenetics underwent ‘continual baseline reprogramming’ [10]. Then, sperm DNA methylation in the F3, F4, and F5 (the first transgenerational generation) exhibited more similarities than did the toxicant exposed F0-F3 generations. And numerous pathologies occurred more frequently owing to F0-F3 toxicant exposure including in the transgenerational epigenetic F5 generation [10]. Moreover, in the review article in this collection [9] citing the preceding work [10], Korolenko and Skinner argue convincingly that transgenerational epigenetic inheritance could contribute significantly to evolution especially in plants and invertebrate animals. However, direct evidence for such mechanisms in mammals remains limited based on the necessary criteria outlined above by Khatib and associates [4].

In this regard, several articles in this collection provide glimpses into epigenetic mechanisms that may underlie transgenerational epigenetic inheritance in mammals and even ways to detect this inheritance in humans. For example, Richards-Steed et al. point out that when proband expressions of adverse phenotypes are associated with shared paternal grandparent environments, transgenerational epigenetic inheritance may have occurred and warrants further study [11]. In another study, Madrid and co-workers report that folate supplementation of F0 mice and rats enhances axon regeneration in un-supplemented F1-F3 progeny following spinal cord injury of the progeny [12]. And using F0 mice – deficient in the gene (*Dio3*) which regulates thyroid hormone sensitivity – Martinez and associates observed altered *Dio3* expression in genetically intact descendants exposed to excess thyroid hormone [13].

In another paper in this collection, however, Sapozhnikov and Szyf [14] provide evidence that a technique used to modify DNA methylation levels also can cause genetic changes in mice. Thus, further emphasizing the difficulties in documenting the occurrence of transgenerational epigenetic inheritance in mammals. They argue that a CRISPR/Cas9-based epigenetic editing method can also cause genetic changes that then produce the heritable epigenetic modifications observed in the study they critique.

In contrast to mammals, transgenerational epigenetic inheritance has been well documented in invertebrate animals [4,5]. In their contribution to this Article Collection, Jeremias et al. [15] further expand this evidence in *Daphnia magna*. They found that toxic copper exposure of F0 animals led to the same modified transcriptional patterns in subsequent F1, F2, and F3 generations. These modifications included increased levels of transcripts of genes involved in DNA repair, mitigation of oxidative stress, detoxification, epigenetic regulation, and functioning of the circadian clock. The latter authors did not, however, demonstrate a clear mechanism for the transgenerational epigenetic inheritance they observed. But in this regard and in another paper in this collection, Doan and associates [16] provide further evidence that non-coding RNA mediates DNA methylation of rat imprinted genes and, by extension, possibly many other epigenetic modifications of DNA and histones [5]. In their study, Doan et al. found that growth restricted rat offspring exhibited imprinted gene alterations in their kidneys that were mediated by long non-coding RNA [16]. Similarly, in a paper in this collection, Champroux and coworkers report that transmission of decreased amounts of miRNA-34/449 from sperm to preimplantation embryos causes anxiety and defective sociability in female offspring of mice and possibly humans [17].

Similar to invertebrate animals, plants exhibit well documented transgenerational epigenetic inheritance [4,5]. In the present article collection, Quan and associates provide data to reenforce and expand this notion [18]. They found that DNA methylation sites in offspring were affected by both the prior parental environment and that of the offspring. Apparently as a result, parents gave rise to progeny that grew in sections containing lead by producing reduced underground biomasses in those contaminated sections – if their parents had not experienced lead contamination. But when parents had experienced this contamination, offspring grew in sections that were not contaminated with lead rather than in those patches where lead was present [18].

Hence, this article collection contributes to our understanding of how epigenetic changes can be transmitted across multiple generations in animals, plants, and other eukaryotic organisms. While transgenerational epigenetic inheritance has been well documented in plants and invertebrate animals [4,5], its further substantiation in vertebrates including humans and other mammals is exciting to anticipate. The importance of such studies especially to the possible transgenerational inheritance of diseases and disorders, like obesity and type 2 diabetes mellitus, cannot be over emphasized. Since toxin exposure gives rise to numerous such afflictions – and these exposures may occur more frequently in less privileged communities – further studies are also needed to understand the social determinants of health and to rectify related public health injustices. And perhaps most promising – in regard to eventually helping people on an individual basis – are findings that some transgenerational epigenetic changes, such as those associated with diet, can be beneficial [7]. Similarly, epigenetic changes not only link adverse childhood experiences with depression, but other epigenetic changes may be protective from such experiences [19].

## Data Availability

Data sharing is not applicable to this article as no new data were created or analysed in this study.
